# Prognostic impact of the controlling nutritional status score in patients with hematologic malignancies: A systematic review and meta-analysis

**DOI:** 10.3389/fimmu.2022.952802

**Published:** 2022-10-05

**Authors:** Chuanyang Lu, Qiuni Chen, Linrong Fei, Junhui Wang, Chunling Wang, Liang Yu

**Affiliations:** ^1^ Department of Hematology, The Affiliated Huaian No.1 People’s Hospital of Nanjing Medical University, Huaian, China; ^2^ Key Laboratory of Hematology, Nanjing Medical University, Nanjing, China; ^3^ The Huaian Clinical College of Xuzhou Medical University, Huaian, China

**Keywords:** controlling nutritional status (CONUT) score, hematologic malignancy, prognosis, overall survival (OS), progression-free survival (PFS)

## Abstract

**Background:**

An increasing number of studies have validated the prognostic significance of the controlling nutritional status (CONUT) score in patients with solid tumors. However, the extent of the correlation between the CONUT score and clinical outcomes of patients with hematologic malignancies is unclear.

**Objective:**

This study aimed to investigate the prognostic role of the CONUT score in patients with hematologic malignancies.

**Methods:**

All relevant articles published up to November 15, 2021, were identified by systematically searching PubMed, Embase, Web of Science, and Cochrane Library. Pooled hazard ratios (HRs) and 95% confidence intervals were used to quantitatively analyze the association between the CONUT scores and clinical outcomes of patients with hematologic malignancies. Subgroup and sensitivity analyses were performed. Funnel plots as well as Begg’s and Egger’s tests were used to assess publication bias.

**Results:**

Six studies with 1811 patients were included in the meta-analysis. The results showed that a high CONUT score was associated with worse overall survival (OS) (HR=1.34, 95%CI 1.14-1.59, P < 0.001) and progression-free survival (PFS) (HR=1.20, 95%CI 1.10-1.32, P < 0.001).

**Conclusions:**

The CONUT score is an independent prognostic factor in patients with hematologic malignancies.

**Systematic review registration:**

http://www.crd.york.ac.uk/prospero/, identifier CRD42021292621.

## Introduction

Hematologic malignancies are a broad group of neoplastic diseases originating from hematopoietic and lymphoid tissues and include various forms of leukemia, malignant lymphomas, and multiple myeloma (MM) (1, 2). New treatment strategies, including targeted therapy, immunotherapy, immune checkpoint inhibitors, chimeric antigen receptor-T cells, and hematopoietic stem cell transplantation have generally improved the OS of patients with hematologic malignancies (3). However, hematologic malignancies rank among the top ten for both morbidity and mortality of malignant tumors in China, and the prognosis for patients with hematologic malignancies tends to be poor.

The CONUT score is an automatic tool for nutritional screening and was initially developed by González-Madroño et al. (4) It is calculated using three variables: serum albumin concentration, cholesterol level, and peripheral lymphocyte count. According to the total scores, the CONUT score is then stratified into four levels: normal (0-1 points); mild (2-4 points); moderate (5-8 points); severe (9-12 points). The scoring criteria for CONUT score is summarized in **Table 1** (5).

**Table 1:** The scoring criteria for the CONUT score.

Factors	Degree			
	Normal	Mild	Moderate	Severe
**Serum albumin(g/dL)**	≥3.50	3.00-3.49	2.50-2.99	<2.50
**Score**	0	2	4	6
**Total lymphocyte count(/ml)**	≥1600	1200-1599	800-1199	<800
**Score**	0	1	2	3
**Total cholesterol(mg/dL)**	≥180	140-179	100-139	<100
**Score**	0	1	2	3

Over the past decade, relevant research has validated the prognostic impact of the CONUT score for solid tumors (6–9). More recently, many studies have explored the association between the CONUT score and prognosis for hematologic malignancies most of which concluded that the CONUT score was an independent prognostic factor in patients with hematologic malignancies. However, the prognostic role of the CONUT score for hematologic malignancies is unclear as results are controversial for different types of hematologic diseases. Liang Fei et al. (10) suggested that a high CONUT score was not associated with worse survival in patients with MM (HR=1.19, 95%CI 0.666-2.129, P=0.556). Therefore, owing to the controversial results from different studies, the present meta-analysis was conducted to systematically investigate the predictive significance of CONUT scores for the clinical outcomes of patients with hematologic malignancies.

## Methods

### Search strategies

A systematic search of PubMed, Embase, Web of Science, and Cochrane Library was performed to screen all articles related to the predictive role of CONUT scores in patients with hematologic malignancies up to November 15, 2021. The following search terms were used in PubMed: “controlling nutritional status”, “CONUT”, “controlling nutritional status score”, “CONUT score”, “leukemia”, “lymphoma”, “myeloma”, “hematologic malignancy”, “hematopoietic malignancy”, “hematopoietic neoplasms”, “hematological malignancy”, “hematological neoplasms”, and “hematologic neoplasms”. The retrieval process was independently conducted by two authors.

### Selection criteria

Following the retrieval of relevant articles using the search terms, articles were then selected based on the following criteria: (i) articles concerning the predictive significance of the CONUT score in patients with hematologic malignancies; (ii) patients were newly diagnosed with a hematologic malignancy and divided into two groups; (iii) the presence of HR and their 95% CI and P-value for OS, PFS or other effect indices; (iv) the study was published in full text.

Additionally, retrieved articles that met the following criteria were excluded from the review: (i) prior review, letter, case report, conference abstract, comment, meta-analysis, book and documents, and unpublished articles; and (ii) studies with sample size less than 100.

Two authors independently evaluated the eligibility of studies based on the above selection criteria and a third author was consulted to resolve any disagreements.

### Data extraction and quality assessment

Critical data were extracted from the selected studies, including the name of the first author, year of publication, country, study design, type of hematologic malignancy, number of patients, cut-off for a high CONUT score, median age of patients, and study endpoints. The Newcastle–Ottawa quality assessment Scale (NOS) was used to evaluate the quality of the included studies (11). Studies with a score greater than or equal to six were included in the meta-analysis.

### Statistical analysis

All data analyses were performed using STATA (version MP 16.0) and Review Manager (version 5.4.1). Cochran’s Q test and the I^2^ index were used to evaluate heterogeneity among the different studies. I^2^ values greater than 50% and P-values less than 0.10 revealed significant heterogeneity among the studies. A random-effects model was used to pool OS and PFS. The pooled HR was considered statistically significant when its P-value was less than 0.05, and the 95% CI did not overlap (12, 13). Sensitivity and subgroup analyses were employed to explore the sources of heterogeneity. Publication bias was estimated using funnel plots, Begg’s test, and Egger’s test.

## Result

**Figure 1:**
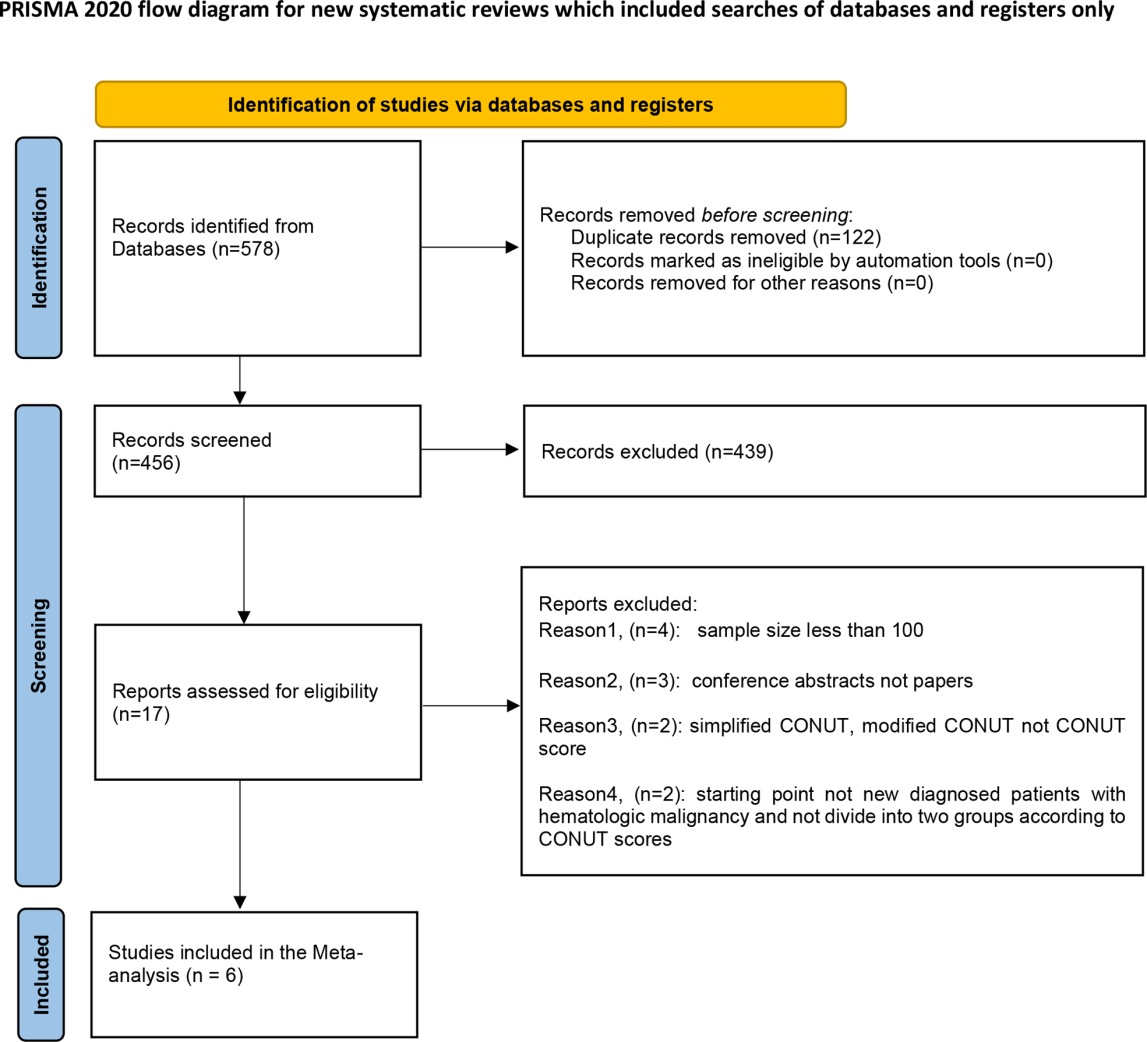
The flow diagram of the search and selection process.

After searching the databases, 578 relevant studies were identified, of which 122 studies were excluded due to duplication. During the screening process, 439 studies were screened out following analysis of the abstracts as these studies did not investigate the prognostic role of CONUT scores in hematologic malignancies. Eleven studies were excluded as they did not fulfill the inclusion criteria. Subsequently, six studies were selected for this meta-analysis. A flow diagram of the search and selection process is shown in **Figure 1**.

The main clinical characteristics of the included studies are summarized in **Table 2**. Within the six studies, 1811 patients from Japan, Turkey, and China were eligible for inclusion. Five of the included studies were single-center studies and only one multicenter retrospective study was included. Hematological disorders mainly included MM and diffuse large B-cell lymphoma (DLBCL). The median age of the patients ranged from 56 to 76 years.

**Table 2:** The characteristics of the included studies.

Author	Year	Country	Study design	Disease	Number	Cut-off	Median age (years)	Endpoints	NOS
**Kamiya** **et al.** (14)	2020	Japan	Retrospective Single-center	MM	178	≥5	CONUT≥5 76 (38-92) CONUT ≤ 4 69 (39-86)	OS, HR=2.364 (1.324-4.220), P=0.004**	8
**Nagata** **et al.** (15)	2020	Japan	Retrospective Single-center	DLBCL	476	≥4	68.5 (27-97)	OS, HR=1.86 (1.24-2.80), P<0.01** PFS, HR=1.42 (0.98-2.06), P=0.06**	6
**Matsukawa** **et al.** (16)	2020	Japan	Retrospective multicenter	DLBCL	615	>4	69 (20-97)	OS, HR=1.53 (1.05-2.23), P=0.028**	7
**Li** **et al.** (17)	2021	China	Retrospective Single-center	MM	119	>3.5	56 (23-83)	OS, HR=1.198 (1.036-1.385), P=0.015**	7
**ÇAĞLIYAN** **et al.** (18)	2021	Turkey	Retrospective Single-center	DLBCL	266	≥2	64 (23-91)	OS, HR=1.15 (1.04-1.26), P=0.003** PFS, HR=1.19 (1.08-1.31), P=0.001**	6
**Liang** **et al.** (10)	2021	China	Retrospective Single-center	MM	157	>3.5	64 (30-91)	OS, HR=1.191 (0.666-2.129), P=0.556**	8

**multivariate analysis; MM, multiple myeloma; DLBCL, diffuse large B cell lymphoma; NOS, the Newcastle–Ottawa quality assessment Scale.

### Prognostic effect of CONUT scores on patients’ OS

**Figure 2:**
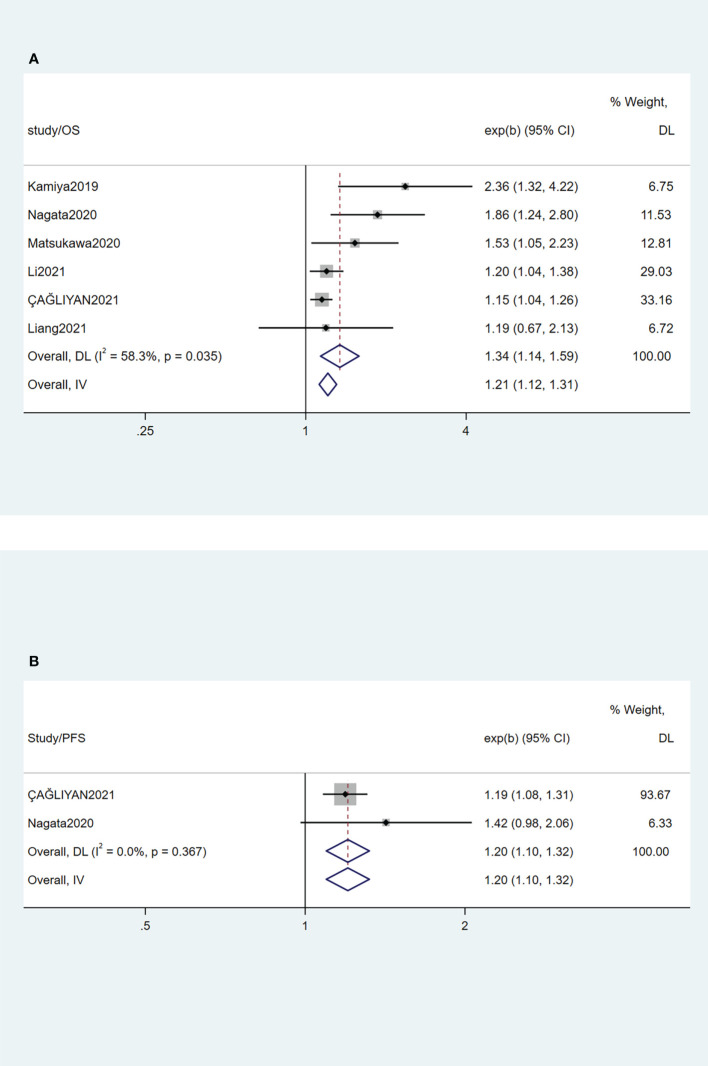
Forest plots for high CONUT score have a detrimental effect on OS **(A)** and PFS **(B)** of patients with hematologic malignancy.

Six studies (10, 14–18) investigated the OS of patients with hematologic malignancy. Following the results of the heterogeneity test with an I^²^ value 58.3 and a P-value of 0.035, the random-effects model was chosen. The pooled HRs, confirmed that a high CONUT score was a risk factor for patients with hematologic malignancy (HR=1.34, 95% CI 1.14-1.59, P=0.035) (**Figure 2A**). It can be concluded that patients with hematologic malignancies and poor nutritional status have an increased risk of mortality.

**Figure 3:**
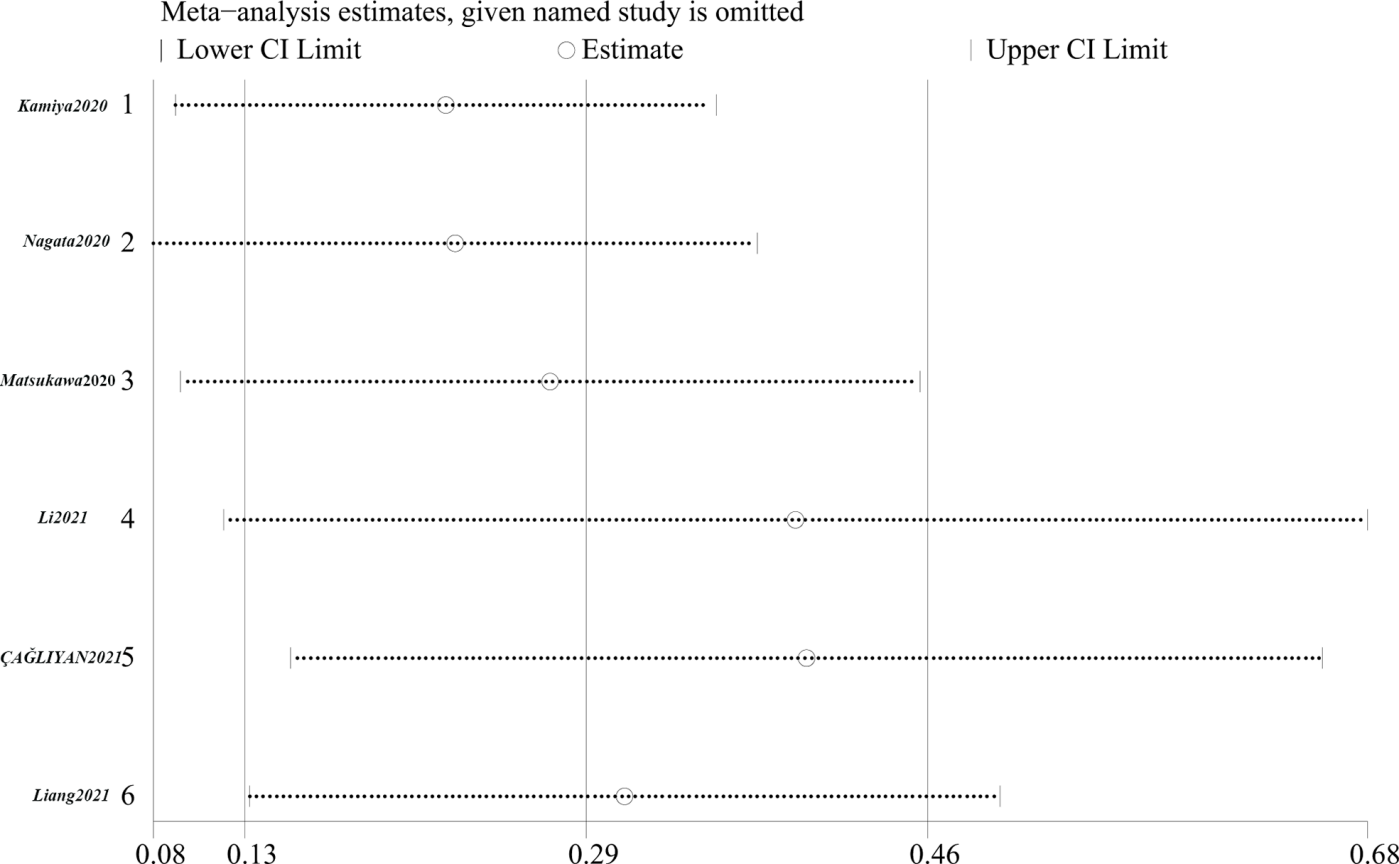
The sensitivity analysis of the prognostic role of high CONUT score on OS.

The results of the sensitivity analysis are shown in **Figure 3**. The pooled lnHR was 0.29 (95% CI 0.13-0.46). After the arbitrary deletion of one study, the pooled lnHRs of the remaining studies ranged from 0.23 to 0.40. Thus, the arbitrary deletion of one piece of literature from this meta-analysis did not affect the results, implying that the above conclusions of the random effects model are stable and reliable.

**Figure 4:**
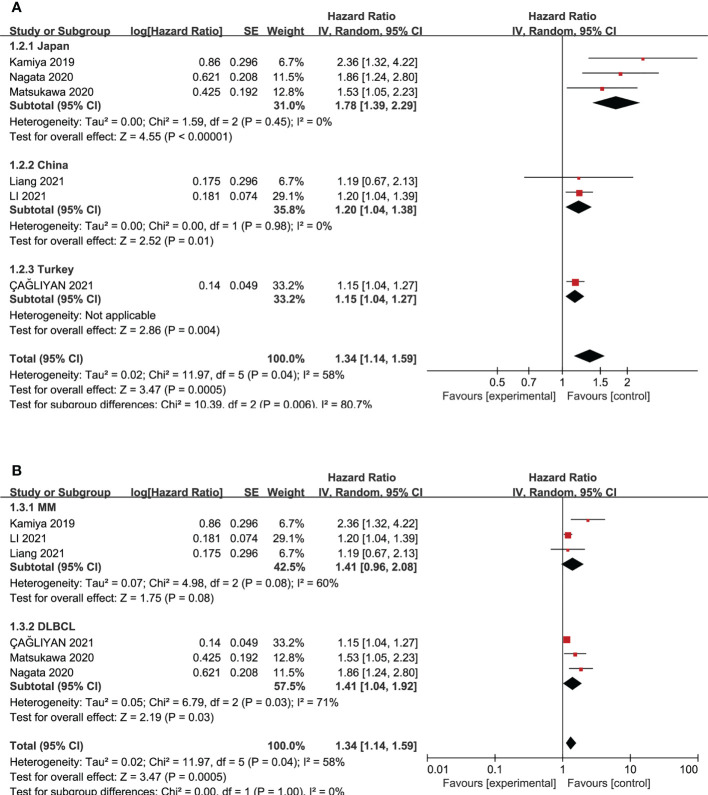
Subgroup analysis of the prognostic role of high CONUT score on OS according to their races **(A)** and types of hematologic malignancies **(B)**.

Stratification by disease type showed that the HR was 1.41 (95% CI 0.96-2.08, I²=60%, P=0.08) for MM and 1.41 (95% CI 1.04-1.92, I^2 =^ 71%, P=0.03) for DLBCL. Stratification by patients’ nationality indicated that the HR was 1.78 (95% CI 1.39-2.29, I^2 =^ 0%, P=0.45), 1.20 (95% CI 1.04-1.38, I^2 =^ 0%, P=0.98), and 1.15 (95% CI 1.04-1.27) for Japan, China, and Turkey, respectively. The results of subgroup analysis are shown in **Figure 4**. From the results, it can be concluded that the heterogeneity of the meta-analysis may originate from patient race and is not related to the disease type.

**Figure 5:**
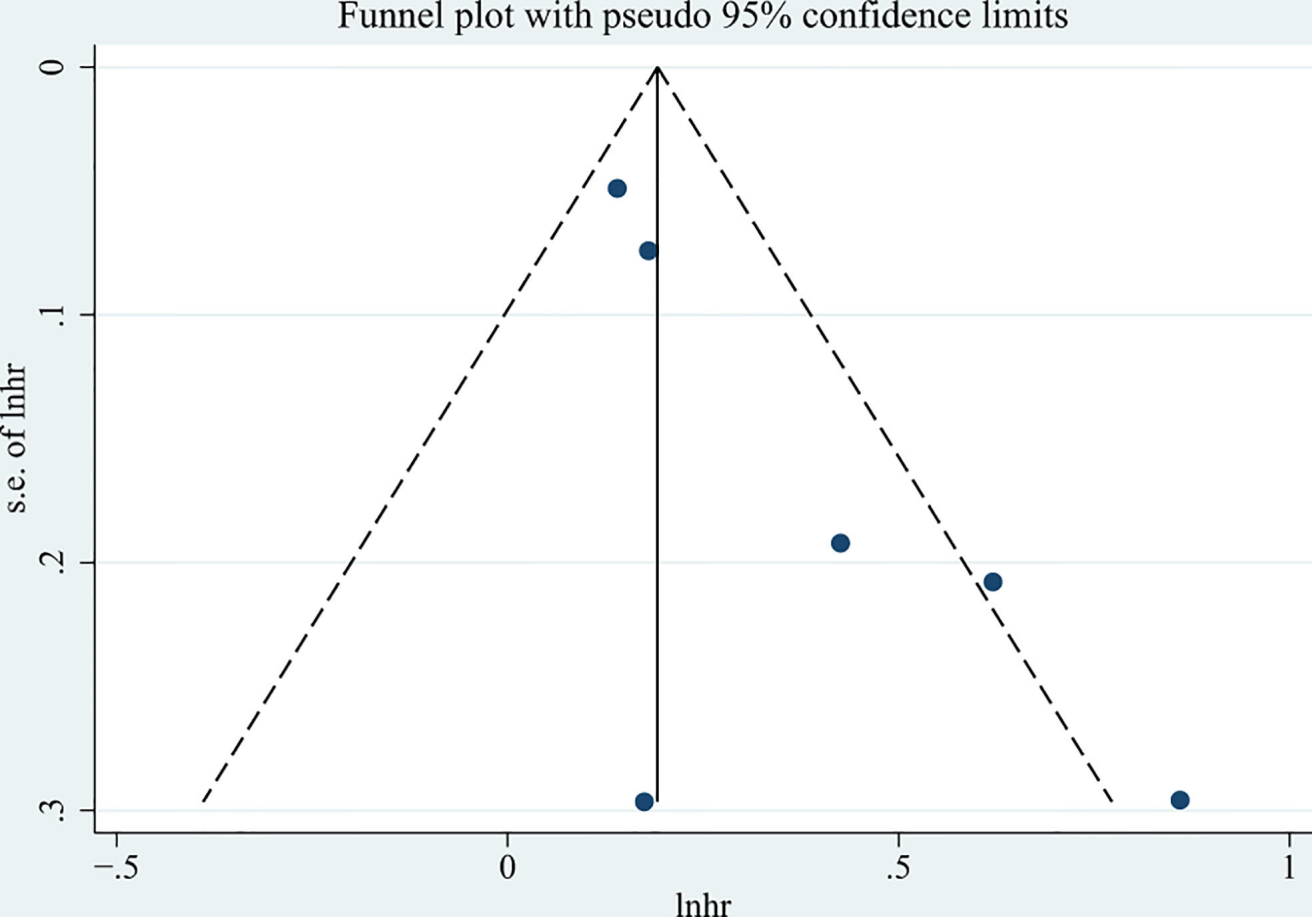
The funnel plot of the six selected studies.

The funnel plot presented in **Figure 5** is asymmetrical. The p-values of the Begg’s and Egger’s tests were 0.133 (> 0.05) and 0.038 (< 0.05), respectively. In combination with funnel plots, Begg’s test, and Egger’s test, it is suggested that the meta-analysis has a certain publication bias.

### Prognostic effect of CONUT scores on patients’ PFS

PFS was researched in two studies with 742 patients. ÇAĞLIYAN A et al. reported that a high CONUT score group was correlated to a worse PFS in patients with DLBCL after univariate analysis (18). Multivariate analysis also showed that the CONUT score was an independent prognostic factor for PFS. Nagata et al. compared PFS between the high CONUT score group and the low CONUT score group, which showed a distinct difference (5-year PFS, 46.1% vs. 73.1%, P < 0.001) (15). However, in the multivariate analysis, the difference was not statistically significant (HR=1.42, 95% CI 0.98-2.06, P=0.06). The results of comparing PFS in the high and low CONUT score groups are shown in **Figure 2B**. The forest plot illustrates that patients in the high CONUT score group had a significantly worse PFS (HR=1.20, 95% CI 1.10-1.32, P < 0.001) than those in the low score group.

## Discussion

Previous studies have focused on the prognostic effect of the CONUT score in patients with solid tumors (19–24). Recently, several studies have retrospectively analyzed the prognostic role of CONUT scores in patients with hematologic malignancies (25–28). Because of the controversial results from different studies, we conducted a meta-analysis to investigate the prognostic value of CONUT score in patients with hematologic neoplasms.

The present meta-analysis concluded that CONUT score is an independent prognostic factor in patients with hematologic malignancies. Patients with high CONUT scores had worse OS and PFS than those with low CONUT scores. The sensitivity analysis confirmed that the results of the meta-analysis were stable and reliable. The results of the subgroup analysis showed substantial heterogeneity between subgroups of patient races, which may be explained by the different demographic characteristics of patients enrolled from different countries. Investigation of racial and demographic effects on these indices could be a priority for future research.

The mechanisms underlying the prognostic role of CONUT score in hematologic malignancies have not been well explored. We can only postulate that each component of the CONUT score is associated with the prognosis of patients with hematological malignancies.

Baseline albumin level can be used as a biomarker to indicate a patient’s overall nutritional and immune status. Moreover, it can be used as a marker of systemic inflammatory responses in cancer (29). Low albumin concentration was positively correlated with poor clinical outcomes in hematological malignancies such as MM, DLBCL and pediatric acute lymphoblastic leukemia (30–32).

Cholesterol is an important component of mammalian cell membranes (33). Contrary to popular thought, studies have shown that low LDL-C levels are correlated with an increased risk of hematological cancer (34). Additionally, there is a significant decrease in total cholesterol levels at the onset of hematologic disease, and continued decrease with progression of the disease (35). The underlying mechanism could be attributed to the growth of neoplastic cells demanding high cholesterol levels (35, 36). It is reasonable to deduce that serum cholesterol levels can be used as a biochemical prognostic marker in patients with hematological malignancies.

Absolute lymphocyte count can indicate the host’s systemic immune status. Many studies have confirmed the prognostic significance of absolute lymphocyte counts in patients with hematological malignancies, such as MM and DLBCL (37, 38). Therefore, peripheral total lymphocyte count can be used as an immune-nutritional biomarker to predict the clinical outcomes of patients with hematologic malignancies.

In general, the CONUT score was started as a nutritional screening tool and is now widely used to evaluate the prognostic role in patients with cancer. Relevant studies have confirmed the higher predicting accuracy of the CONUT score compared to other nutrition indicators such as neutrophil to lymphocyte ratio (NLR), platelet to lymphocyte ratio (PLR), and prognostic nutritional index (PNI). The possible reasons for interpreting the association between high CONUT scores and poor long-term outcomes can be summarized as follows: malnutrition in patients with tumors causes decreased tolerance to chemotherapy and increased incidence of chemotherapy-related toxicity, leading to the failure of the treatment regimens and affecting the prognosis of patients with cancer. On the other hand, the CONUT score is significantly associated with inflammation. Inflammation might lead to bone marrow failure or leukemia, promote myeloid malignancy progression, and exacerbate symptom burden.

This study aimed to validate the prognostic impact of CONUT scores in patients with hematologic malignancies. Using these three parameters, we can identify patients with poor nutritional status at diagnosis. Based on this, individualized nutritional supplement protocols can be developed to actively correct malnutrition and immune system disorders to improve patient prognosis (10).

The present meta-analysis has several limitations. All included studies were retrospective cohort studies, and five of the six studies were single-center studies. In addition, the p-value of Begg’s test was 0.133 and Egger’s test was 0.038 (less than 0.05), which suggested that this meta-analysis may have some degree of publication bias. Therefore, prior to translating the results of this meta-analysis into clinical application, further multicenter prospective cohort studies with large sample sizes are needed to investigate the prognostic role of CONUT scores in the long-term outcomes of patients with hematologic malignancies.

## Conclusion

From the above meta-analysis, it can be concluded that CONUT score is an independent prognostic factor in patients with hematologic malignancies. Malnourished patients were associated with lower OS and PFS. As a convenient and objective nutritional screening tool, the CONUT score can optimize risk stratification to guide physicians in selecting treatment modalities and judging prognosis in their daily clinical practice. More studies are needed to validate the significance of the CONUT score in patients with hematological malignancies.

## Data availability statement

The original contributions presented in the study are included in the article/supplementary material. Further inquiries can be directed to the corresponding author.

## Author contributions

QC contributed to conception and design of the study. CL and QC were responsible for searching the database and CL wrote the first draft. LF and JW were responsible for the creation of tables and figures. LY revised the manuscript and was in charge of the communication. Particularly, CW provided professional guidance and made great contributions in the process of manuscript revise. All authors contributed to the article and approved the submitted version.

## Funding

This work was funded by the Science and Technology Fund of Huaian City [grant # HAB202020] and the Commission of Health of Jiangsu Province [grant # 2019082].

## Conflict of interest

The authors declare that the research was conducted in the absence of any commercial or financial relationships that could be construed as a potential conflict of interest.

## Publisher’s note

All claims expressed in this article are solely those of the authors and do not necessarily represent those of their affiliated organizations, or those of the publisher, the editors and the reviewers. Any product that may be evaluated in this article, or claim that may be made by its manufacturer, is not guaranteed or endorsed by the publisher.
